# Safely correct hyponatremia with continuous renal replacement therapy: A flexible, all‐purpose method based on the mixing paradigm

**DOI:** 10.14814/phy2.15496

**Published:** 2023-01-05

**Authors:** Sheldon Chen, Jerry Yee, Robert Chiaramonte

**Affiliations:** ^1^ Section of Nephrology MD Anderson Cancer Center Houston Texas USA; ^2^ Division of Nephrology and Hypertension Henry Ford Hospital Detroit Michigan USA; ^3^ Internal Medicine SUNY Downstate New York New York USA

**Keywords:** CRRT, hemofiltration, kinetics, mathematical models

## Abstract

Treating chronic hyponatremia by continuous renal replacement therapy (CRRT) is challenging because the gradient between a replacement fluid's [sodium] and a patient's serum sodium can be steep, risking too rapid of a correction rate with possible consequences. Besides CRRT, other gains and losses of sodium‐ and potassium‐containing solutions, like intravenous fluid and urine output, affect the correction of serum sodium over time, known as osmotherapy. The way these fluids interact and contribute to the sodium/potassium/water balance can be parsed as a mixing problem. As Na/K/H_2_O are added, mixed in the body, and drained via CRRT, the net balance of solutes must be related to the change in serum sodium, expressible as a differential equation. Its solution has many variables, one of which is the sodium correction rate, but all variables can be evaluated by a root‐finding technique. The mixing paradigm is proved to replicate the established equations of osmotherapy, as in the special case of a steady volume. The flexibility to solve for any variable broadens our treatment options. If the pre‐filter replacement fluid cannot be diluted, then we can compensate by calculating the CRRT blood flow rate needed. Or we can deduce the infusion rate of dextrose 5% water, post‐filter, to appropriately slow the rise in serum sodium. In conclusion, the mixing model is a generalizable and practical tool to analyze patient scenarios of greater complexity than before, to help doctors customize a CRRT prescription to safely and effectively reach the serum sodium target.

## Graphical abstract

The treatment of chronic hyponatremia by CRRT is complicated, but it can be modeled as a calculus mixing problem. The differential equation's solution corroborates the science of osmotherapy and shows us how to use the various CRRT settings to safely correct hyponatremia.

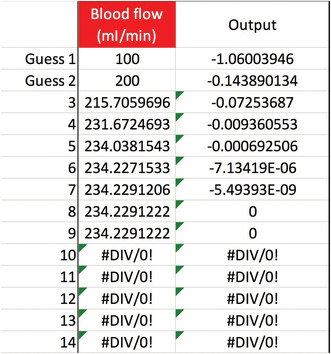

## 1 INTRODUCTION

Chronic hyponatremia, defined as a low serum sodium for more than 48 h, is a vexing problem during renal replacement therapy (RRT). The low sodium needs to be raised slowly to avoid osmotic demyelination syndrome (King & Rosner, 2010), and the guidelines suggest a goal for correction of 4–6 mEq/L per day but no more than 8–12 mEq/L per day (Spasovski et al., 2014; Verbalis et al., 2013). On the other hand, RRT can raise the serum sodium much faster than the recommended rate. The serum sodium will tend to equilibrate with the sodium concentration in the dialysate or replacement fluid (RF) that is generally around 140 mEq/L. To balance these competing rates, nephrologists purposely dilute the dialysate or RF [sodium], both to attenuate the rate of correction and to cap the serum sodium at a level that complies with the hyponatremia guidelines (Bender, 1998; Dangoisse et al., 2014; Hasegawa et al., 2016; Viktorsdottir et al., 2013). The dilution should be determined scientifically, based upon the principles of sodium kinetics. This field uses sophisticated modeling to predict how the serum sodium will evolve under various perturbations, including RRT (Mohiuddin et al., 2022; Yee et al., 2020; Yessayan et al., 2016; Yessayan et al., 2021). Its quantitative underpinnings allow us to calculate ways to gradually increase the serum sodium throughout an RRT session. The goal is to treat chronic hyponatremia in a more precise and rational way that emphasizes safety.

### 1.1 Mixing problem

The downside of sophistication is complexity. Most physicians will not know how to use sodium kinetics to prescribe a CRRT to treat hyponatremia. But maybe they will consult the technique more often if the underlying math is done by software. We decided to model the treatment of chronic hyponatremia by RRT as a “mixing problem,” as taught in calculus. A typical problem might be, “A 100‐gallon tank is 90% full with water. A liquid dye is pumped into the tank at one gallon/minute. The mixture is stirred well and drained at a rate of half a gallon/minute. What is the concentration of dye when the tank is full?” (Answer: 19%; see Appendix A.) Adapting this construct to hyponatremic patients getting RRT, we could ask, “If a patient with a total body water (TBW) of 48 liters and a serum sodium of 116 mEq/L is treated with continuous venovenous hemofiltration (CVVH) such that the blood flow rate $\left(Q_{B}\right)$ is 200 ml/min, the ultrafiltration (UF) rate is 42 ml/h, and the pre‐filter (or pre‐dilution) RF rate is 1200 ml/h, what should the RF's $\left[\mathrm{Na}+K\right]$ be diluted to in order to increase the serum sodium by 8 mEq/L in 24 hours?” (Answer: 135 mEq/L; see p. 5.)

## 2 MATERIALS AND METHODS

### 2.1 Human tank

Instead of the 100‐gallon tank above, the human body can be conceived of as a TBW‐liter tank. Dissolved in that water are sodium and potassium ions such that their quantity divided by the TBW is linearly proportional to the serum sodium concentration, according to the Edelman et al. equation: $\left[\mathrm{Na}\right]=1.11\cdot \frac{\mathrm{Na}+K}{\mathrm{TBW}}-25.6$ (Edelman et al., 1958). If the general equation for a line is y=mx+b, then the independent variable x is $\frac{\mathrm{Na}+K}{\mathrm{TBW}}$, and the dependent variable y is $\left[\mathrm{Na}\right]$. Also, the slope m is 1.11, and the y‐intercept b is −25.6. These values are controversial (Nguyen et al., 2016; Nguyen & Kurtz, 2004a), so until better measurements are made, it may be prudent to just denote them as m and b, as in $\left[\mathrm{Na}\right]=m\cdot \frac{\mathrm{Na}+K}{\mathrm{TBW}}+b$. Most sodium equations assume m=1 and b=0 (Chen, 2019; Chen et al., 2021; Chen & Shey, 2018; Mohiuddin et al., 2022); these values are used throughout, except where noted. Note: m is a scalar (unitless), and b has units of mEq/L.

### 2.2 Strategy for differential equation

The differential equation for a mixing problem is based on the conservation of mass. In the case of the dysnatremias, the relevant mass is the sodium+potassium quantity (Nguyen & Kurtz, 2004b; Shah & Bhave, 2018):

(1) $$\left[\mathrm{Na}\right]=m\cdot \frac{\mathrm{Na}+K}{\mathrm{TBW}}+b\to \mathrm{Na}+K=\frac{\mathrm{TBW}}{m}\cdot \left(\left[\mathrm{Na}\right]-b\right).$$

The rate of change in the Na+K equals the rate of all the Na+K being pumped in minus the rate of all the Na+K being drained out of the human tank, to continue with the mixing analogy. The actual Na+K quantity at any point in time is given by:

(2) $${\left(\mathrm{Na}+K\right)}_{t}=\frac{{\mathrm{TBW}}_{t}}{m}\cdot \left({\left[\mathrm{Na}\right]}_{t}-b\right),$$

where the t subscripts indicate that the variable is now a function of time. The TBW varies over time because intravenous (IV) fluids are infused, urine is produced, and UF is occurring, to list a few inputs and outputs. Of course, we want the serum sodium to vary over time (at a safe rate), and ultimately we are going to solve the differential equation for ${\left[\mathrm{Na}\right]}_{t}$.

### 2.3 Drain out

Pretend that the bottom of the human tank where the well‐mixed Na/K solution is being drained is represented by the arterial port that is taking blood out of the body to be processed by CVVH. While blood is still in the tubing, technically no sodium or potassium or water has been drained. Once the blood reaches the pre‐filter part of the CVVH circuit, it is mixed with the RF. If instant mixing is assumed, then the blood in the circuit has a new concentration. To find it, define the relevant variables as follows: (1) ambient serum sodium concentration: ${\left[\mathrm{Na}\right]}_{t}$, (2) blood flow rate: $Q_{B}$, (3) pre‐filter RF sodium+potassium concentration: ${\left[\mathrm{Na}+K\right]}_{\mathrm{Pre}-\mathrm{RF}}$, and (4) pre‐filter RF flow rate: $Q_{\mathrm{Pre}-\mathrm{RF}}$. So, blood mixed with pre‐filter RF changes the concentration of the sodium to:
(3)

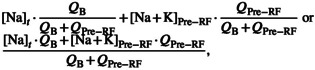

which is an average of the serum [sodium] and the RF's $\left[\mathrm{Na}+K\right]$, weighted by their flow rates.

Upon reaching the filter, the mixture of blood and pre‐RF is filtered out of the circuit as effluent. Usually, CVVH removes as much effluent volume as the added pre‐RF volume, and then some. Any extra effluent is considered to be net ultrafiltration. Incorporate the element of time to turn these volumes into rates, so that the effluent rate is $Q_{\mathrm{Eff}}$ and the UF rate is $Q_{\mathrm{UF}}$. We are now able to express the rate of Na/K mass being drained as:

(4) $$\frac{{\left[\mathrm{Na}\right]}_{t}\cdot Q_{B}+{\left[\mathrm{Na}+K\right]}_{\mathrm{Pre}-\mathrm{RF}}\cdot Q_{\mathrm{Pre}-\mathrm{RF}}}{Q_{B}+Q_{\mathrm{Pre}-\mathrm{RF}}}\cdot Q_{\mathrm{Eff}}.$$

In general, $Q_{\mathrm{Eff}}=Q_{\mathrm{Pre}-\mathrm{RF}}+Q_{\mathrm{UF}}$. However, the net UF rate can be negative, as when CVVH is operated to give volume to a patient, i.e., $Q_{\mathrm{Pre}-\mathrm{RF}}>Q_{\mathrm{Eff}}$ (Macedo & Mehta, 2016; Neyra & Tolwani, 2021; Prowle & Mehta, 2021).

### 2.4 Pump in

One source of Na/K being pumped into the human tank was already discussed above. The pre‐filter RF adds sodium and potassium at the rate of ${\left[\mathrm{Na}+K\right]}_{\mathrm{Pre}-\mathrm{RF}}\cdot Q_{\mathrm{Pre}-\mathrm{RF}}$. To accommodate the practice of running CVVH with both pre‐ and post‐filter RFs, we can describe the post‐filter RF contribution rate as ${\left[\mathrm{Na}+K\right]}_{\text{Post}-\mathrm{RF}}\cdot Q_{\text{Post}-\mathrm{RF}}$. In that case, the total net UF rate due to CVVH is:

(5) $$\begin{array}{c} Q_{\mathrm{UF}}=Q_{\mathrm{Eff}}-Q_{\mathrm{Pre}-\mathrm{RF}}-Q_{\text{Post}-\mathrm{RF}}\to \\ Q_{\mathrm{Eff}}=Q_{\mathrm{Pre}-\mathrm{RF}}+Q_{\text{Post}-\mathrm{RF}}+Q_{\mathrm{UF}}. \end{array}$$

#### 2.4.1 Generic Na/K solutes pump in and drain out

To make the model as versatile as reasonable, we can add in a generic input and a generic output of Na/K solutes. Most hospitalized patients are going to receive IV fluids, which may or may not contain Na/K. We represent that contribution as ${\left[\mathrm{Na}+K\right]}_{\text{In}}\cdot Q_{\text{In}}$. Assume these variables are constants, at least for the duration of the time to treat the hyponatremia. But if an IV fluid is changed, a new calculation can be started. The changes may occur quickly and frequently.

Likewise, some patients getting CVVH could make urine that contains Na/K. Let us represent that drainage rate as ${\left[\mathrm{Na}+K\right]}_{\text{Out}}\cdot Q_{\text{Out}}$. We expect this to have the opposite sign of the inputs but otherwise to behave similarly. Unlike in typical mixing problems, the urine is not just a drainage of the well‐mixed tank fluid. Rather, urine has a composition that is different from ambient ${\left[\mathrm{Na}\right]}_{t}$. Assume the urinary variables are constant, even though the urine concentration is changing over time. These fluctuations can be dealt with using a urinary $\left[\mathrm{Na}+K\right]$ average, which is measured in the laboratory by default since the urine from both kidneys are cumulatively mixed in the bladder.

When we obtain the solution to the differential equation, these generic expressions will show us how to attach more “modules” to account for as many inputs and outputs as are known. The ones that are hard to incorporate (due to lack of data) include stool, sweat, and insensible losses, even though they are as relevant as urine and IV fluids. Other relevant inputs include oral intake, tube feeds, and total parenteral nutrition. The latter two may have $\left[\mathrm{Na}+K\right]$ and rate data in the chart.

### 2.5 Volumes pump in and drain out

We have to keep track of all of the volumes that are going into and out of the human tank. While there is no conservation of volume law, we can pretend that volumes are additive and subtractive, because all of the fluids involved are aqueous. If so, the TBW as a function of time is:

(6) $$\begin{array}{c} {\mathrm{TBW}}_{t}={\mathrm{TBW}}_{0}\\ \;+\left(Q_{\mathrm{Pre}-\mathrm{RF}}+Q_{\text{Post}-\mathrm{RF}}+Q_{\text{In}}-Q_{\mathrm{Eff}}-Q_{\text{Out}}\right)\cdot t \end{array}$$

where ${\mathrm{TBW}}_{0}$ is the initial total body water at time zero. To display the UF rate, we can substitute in the expression for $Q_{\mathrm{Eff}}$, given by Equation (5), and obtain:

(7) $${\mathrm{TBW}}_{t}={\mathrm{TBW}}_{0}+\left(Q_{\text{In}}-Q_{\mathrm{UF}}-Q_{\text{Out}}\right)\cdot t$$

keeping in mind that $Q_{\mathrm{UF}}$ is sometimes negative.

### 2.6 Differential equation: First order, linear

Finally, we are ready to set up a differential equation describing sodium kinetics in CVVH. On the left side of the equation is the derivative of the Na+K function with respect to time. On the right side of the equation are all of the rates of Na+K solutes being pumped in and drained out:

(8) $$\begin{array}{c} \frac{d}{dt}\left[\frac{{\mathrm{TBW}}_{t}}{m}\cdot \left({\left[\mathrm{Na}\right]}_{t}-b\right)\right]={\left[\mathrm{Na}+K\right]}_{\mathrm{Pre}-\mathrm{RF}}\cdot Q_{\mathrm{Pre}-\mathrm{RF}}+{\left[\mathrm{Na}+K\right]}_{\text{Post}-\mathrm{RF}}\cdot Q_{\text{Post}-\mathrm{RF}}\\ \;+{\left[\mathrm{Na}+K\right]}_{\text{In}}\cdot Q_{\text{In}}-\frac{{\left[\mathrm{Na}\right]}_{t}\cdot Q_{B}+{\left[\mathrm{Na}+K\right]}_{\mathrm{Pre}-\mathrm{RF}}\cdot Q_{\mathrm{Pre}-\mathrm{RF}}}{Q_{B}+Q_{\mathrm{Pre}-\mathrm{RF}}}\cdot Q_{\mathrm{Eff}}-{\left[\mathrm{Na}+K\right]}_{\text{Out}}\cdot Q_{\text{Out}}. \end{array}$$

Substituting in for ${\mathrm{TBW}}_{t}$ and $Q_{\mathrm{Eff}}$, our working equation becomes:

(9) $$\begin{array}{c} \frac{d}{dt}\left[\frac{{\mathrm{TBW}}_{0}+\left(Q_{\text{In}}-Q_{\mathrm{UF}}-Q_{\text{Out}}\right)\cdot t}{m}\cdot \left({\left[\mathrm{Na}\right]}_{t}-b\right)\right]={\left[\mathrm{Na}+K\right]}_{\mathrm{Pre}-\mathrm{RF}}\cdot Q_{\mathrm{Pre}-\mathrm{RF}}\\ +{\left[\mathrm{Na}+K\right]}_{\text{Post}-\mathrm{RF}}\cdot Q_{\text{Post}-\mathrm{RF}}+{\left[\mathrm{Na}+K\right]}_{\text{In}}\cdot Q_{\text{In}}-\frac{{\left[\mathrm{Na}\right]}_{t}\cdot Q_{B}+{\left[\mathrm{Na}+K\right]}_{\mathrm{Pre}-\mathrm{RF}}\cdot Q_{\mathrm{Pre}-\mathrm{RF}}}{Q_{B}+Q_{\mathrm{Pre}-\mathrm{RF}}}\cdot \left(Q_{\mathrm{Pre}-\mathrm{RF}}+Q_{\text{Post}-\mathrm{RF}}+Q_{\mathrm{UF}}\right)-{\left[\mathrm{Na}+K\right]}_{\text{Out}}\cdot Q_{\text{Out}}. \end{array}$$

This differential equation can be solved with the standard methods of calculus (see Appendix A). Before showing the full‐fledged answer, in the interest of simplicity, we first show the solution to Equation (9) under conditions of CRRT alone, with no extra inputs or outputs:

$$\begin{array}{c} {\left[\mathrm{Na}\right]}_{t}={\left[\mathrm{Na}\right]}_{0}+\left\{1-{\left[\frac{{\mathrm{TBW}}_{0}}{{\mathrm{TBW}}_{0}+\left(-Q_{\mathrm{UF}}\right)\cdot t}\right]}^{\left[1+m\cdot \frac{Q_{B}\cdot \left(Q_{\mathrm{Pre}-\mathrm{RF}}+Q_{\text{Post}-\mathrm{RF}}+Q_{\mathrm{UF}}\right)}{\left(Q_{B}+Q_{\mathrm{Pre}-\mathrm{RF}}\right)\cdot \left(-Q_{\mathrm{UF}}\right)}\right]}\right\}\cdot \\ \left\{\left[m\cdot \left(\begin{array}{c} {\left[\mathrm{Na}+K\right]}_{\mathrm{Pre}-\mathrm{RF}}\cdot Q_{\mathrm{Pre}-\mathrm{RF}}\cdot \left(1-\frac{Q_{\mathrm{Pre}-\mathrm{RF}}+Q_{\text{Post}-\mathrm{RF}}+Q_{\mathrm{UF}}}{Q_{B}+Q_{\mathrm{Pre}-\mathrm{RF}}}\right)\\ +{\left[\mathrm{Na}+K\right]}_{\text{Post}-\mathrm{RF}}\cdot Q_{\text{Post}-\mathrm{RF}} \end{array}\right)+b\cdot \left(-Q_{\mathrm{UF}}\right)\right]\cdot \frac{Q_{B}+Q_{\mathrm{Pre}-\mathrm{RF}}}{\left(Q_{B}+Q_{\mathrm{Pre}-\mathrm{RF}}\right)\cdot \left(-Q_{\mathrm{UF}}\right)+m\cdot Q_{B}\cdot \left(Q_{\mathrm{Pre}-\mathrm{RF}}+Q_{\text{Post}-\mathrm{RF}}+Q_{\mathrm{UF}}\right)}-{\left[\mathrm{Na}\right]}_{0}\right\} \end{array}$$

For comparison, the most general solution to Equation (9) that allows for multiple inputs and outputs is:

(10) $$\begin{array}{c} {\left[\mathrm{Na}\right]}_{t}={\left[\mathrm{Na}\right]}_{0}+\left\{1-{\left[\frac{{\mathrm{TBW}}_{0}}{{\mathrm{TBW}}_{0}+\left(Q_{\text{In}}\cdots -Q_{\mathrm{UF}}-Q_{\text{Out}}\cdots \right)\cdot t}\right]}^{\left[1+m\cdot \frac{Q_{B}\cdot \left(Q_{\mathrm{Pre}-\mathrm{RF}}+Q_{\text{Post}-\mathrm{RF}}+Q_{\mathrm{UF}}\right)}{\left(Q_{B}+Q_{\mathrm{Pre}-\mathrm{RF}}\right)\cdot \left(Q_{\text{In}}\cdots -Q_{\mathrm{UF}}-Q_{\text{Out}}\cdots \right)}\right]}\right\}\cdot \\ \left\{\left[m\cdot \left(\begin{array}{c} {\left[\mathrm{Na}+K\right]}_{\mathrm{Pre}-\mathrm{RF}}\cdot Q_{\mathrm{Pre}-\mathrm{RF}}\cdot \left(1-\frac{Q_{\mathrm{Pre}-\mathrm{RF}}+Q_{\text{Post}-\mathrm{RF}}+Q_{\mathrm{UF}}}{Q_{B}+Q_{\mathrm{Pre}-\mathrm{RF}}}\right)\\ +{\left[\mathrm{Na}+K\right]}_{\text{Post}-\mathrm{RF}}\cdot Q_{\text{Post}-\mathrm{RF}}\\ +{\left[\mathrm{Na}+K\right]}_{\text{In}}\cdot Q_{\text{In}}\cdots \\ -{\left[\mathrm{Na}+K\right]}_{\text{Out}}\cdot Q_{\text{Out}}\cdots \end{array}\right)+b\cdot \left(Q_{\text{In}}\cdots -Q_{\mathrm{UF}}-Q_{\text{Out}}\cdots \right)\right]\right.\cdot \\ \left.\frac{Q_{B}+Q_{\mathrm{Pre}-\mathrm{RF}}}{\left(Q_{B}+Q_{\mathrm{Pre}-\mathrm{RF}}\right)\cdot \left(Q_{\text{In}}\cdots -Q_{\mathrm{UF}}-Q_{\text{Out}}\cdots \right)+m\cdot Q_{B}\cdot \left(Q_{\mathrm{Pre}-\mathrm{RF}}+Q_{\text{Post}-\mathrm{RF}}+Q_{\mathrm{UF}}\right)}-{\left[\mathrm{Na}\right]}_{0}\right\} \end{array}$$

In the above, an extra variable is ${\left[\mathrm{Na}\right]}_{0}$, the starting serum sodium, introduced when using initial conditions to solve the differential equation. In the various parentheses, the ellipses signify the modular nature of the generic ins and outs. Add as many modules as there are inputs (oral intake, IV fluids, etc.) and outputs (urine, diarrhea, etc.). Finally, when using Equation (10), include the unit conversions if the variables are in different units. For example, the blood flow rate $Q_{B}$ may be in ml/min, but most of the other rates are usually in ml/h.

### 2.7 If TBW is stable

CVVH can be used to maintain a patient's volume at a steady level (keep even). In Equation (7), all of the inputs must be equal to all of the outputs, and then $\left(Q_{\text{In}}-Q_{\mathrm{UF}}-Q_{\text{Out}}\right)=0$. Plugging this into Equation (10) is straightforward except for the exponential in the first line. That term becomes an indeterminate $1^{\infty }$ that, fortunately, can be resolved using limits:
(11)

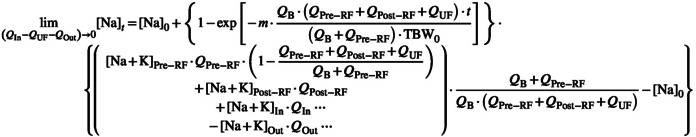

where exp means raise the base e to the exponent shown, making sure that all of its units cancel. The three variables $Q_{\text{In}}$, $Q_{\mathrm{UF}}$, and $Q_{\text{Out}}$ still appear individually, but collectively they must fulfill $Q_{\text{In}}-Q_{\mathrm{UF}}-Q_{\text{Out}}=0$. The simplest case is to have all three variables equal zero.

### 2.8 Computing the variables

Using Equation (10) or (11), we can calculate the serum sodium at a certain time, ${\left[\mathrm{Na}\right]}_{t}$, but that is not useful. Clinically, doctors have an idea of the ${\left[\mathrm{Na}\right]}_{t}$ to aim for, based on the rate of sodium correction guidelines. They want to know the set of conditions that will hit the target. If ${\left[\mathrm{Na}\right]}_{t}$ is pre‐specified, and all the variables (except one) are either measured or assigned, can the missing variable be calculated? A few of the variables cannot be solved for algebraically. At least they can be estimated very accurately using a root‐finding technique. The typical one is Newton's method, but that entails differentiating the function further, which is cumbersome. A simpler technique is the secant method, an iterative process that gives the next x value as: $x_{n+1}=x_{n}-f\left(x_{n}\right)\cdot \frac{x_{n}-x_{n-1}}{f\left(x_{n}\right)-f\left(x_{n-1}\right)}$. Supply initial guesses for $x_{n}$ and $x_{n-1}$, and the iterations will stop when the denominator's two $f\left(x\right)$'s are so close in value (both nearing zero) that the effect is to divide by zero. Then, we can estimate any of the variables in Equation (10) or (11) once it is rearranged to equal zero by moving ${\left[\mathrm{Na}\right]}_{t}$ to the right side of the equation.

## 3 RESULTS

### 3.1 Solving the introduction example

**FIGURE 1:**
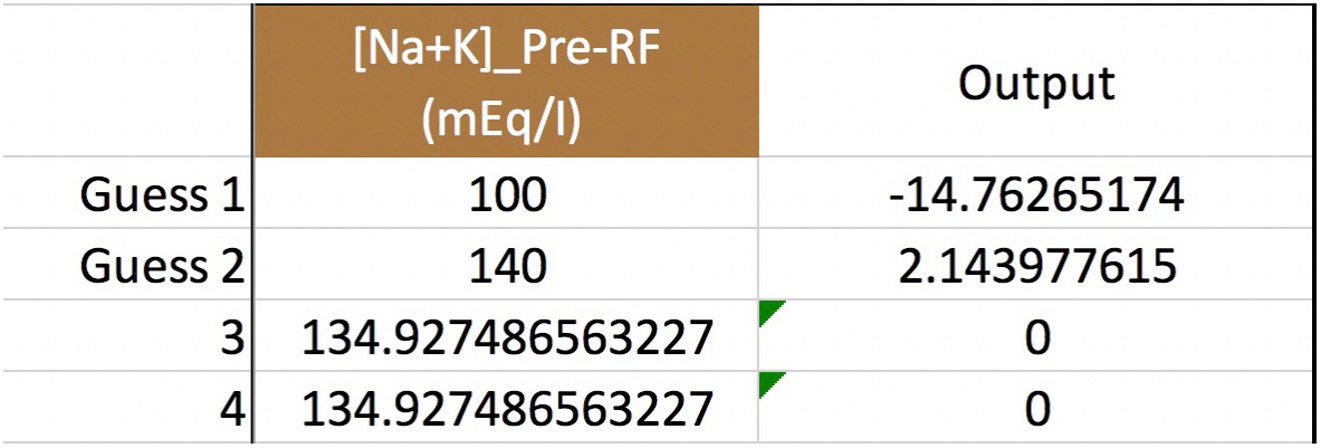
Introduction case: Using the secant method to calculate pre‐filter RF $\left[\mathrm{Na}+K\right]$. In the provided spreadsheet, enter the clinical data given on p. 5, in the requested units. Provide two guesses for ${\left[\mathrm{Na}+K\right]}_{\mathrm{Pre}-\mathrm{RF}}$, and the secant method will converge on the value of ~135 mEq/L. The answer is obtained immediately after the guesses, because each of the $\left[\mathrm{Na}+K\right]$ variables can be solved for algebraically.

Let us use Equation (10) and the secant method to solve the CVVH mixing problem posed in the Introduction. The known values are ${\mathrm{TBW}}_{0}=48$ L, ${\left[\mathrm{Na}\right]}_{0}=116$ mEq/L, ${\left[\mathrm{Na}\right]}_{t}=124$ mEq/L, t=24 h, $Q_{B}=200$ ml/min, $Q_{\mathrm{UF}}=42$ ml/h, and $Q_{\mathrm{Pre}-\mathrm{RF}}=1,200$ ml/h. The other variables are not used $\left(\mathrm{all}=0\right)$. Assume m=1 and b=0 mEq/L. Find the value of ${\left[\mathrm{Na}+K\right]}_{\mathrm{Pre}-\mathrm{RF}}$. This *can* be solved for algebraically, but we will use the secant method anyway. The answer, to an unrealistically accurate degree, is 134.927486563227… mEq/L, the same as would be obtained algebraically (Figure 1). How can we dilute the RF's $\left[\mathrm{Na}+K\right]$ to 135 mEq/L? Say we are using a brand of RF with $\left[\mathrm{Na}\right]=140$ and $\left[K\right]=4$ mEq/L (plus other ingredients). To each 5‐liter RF bag, add about 333 ml of sterile water to dilute the $\left[\mathrm{Na}+K\right]$ of 144 down to 135 mEq/L, as calculated by $\frac{144\;\frac{\mathrm{mEq}}{L}\cdot 5\;L}{\left(5+x\right)\;L}=135\;\frac{\mathrm{mEq}}{L}⟹x=0.\bar{3}$ L. All other RF ingredients are diluted by the same percentage, in this case 93.7%.

### 3.2 Gold standard

How do we know that Equations (10) and (11) are correct in the first place? We can compare their answers to the ones calculated by the more established equations in the field of sodium kinetics. Called osmotherapy, it aims to correct the serum sodium in a gradual, controlled way during continuous dialysis (Yee et al., 2020). How would its equations answer the clinical problem above? First, it equates the [sodium] adjustment ratio (NaAR) to be the urea reduction ratio (URR). Say that the blood urea nitrogen (BUN) goes from 80 to 48 mg/dL for a urea reduction ratio of $\frac{80-48}{80}=0.4$. Then, the 0.4 as the NaAR is used to calculate the $\left[\mathrm{Na}+K\right]$ of the pre‐filter RF: ${\left[\mathrm{Na}+K\right]}_{\mathrm{Pre}-\mathrm{RF}}={\left[\mathrm{Na}\right]}_{0}+\frac{{\left[\mathrm{Na}\right]}_{t}-{\left[\mathrm{Na}\right]}_{0}}{\text{NaAR}}=116+\frac{124-116}{0.4}=136$ mEq/L. Next, calculate the dialysance of sodium: $D_{\mathrm{Na}}=-\frac{{\mathrm{TBW}}_{0}}{t}\cdot \ln\left(1-\text{NaAR}\right)=-\frac{48}{24}\cdot \ln\left(1-0.4\right)\approx 1.02\;\frac{L}{h}=17\;\frac{\text{ml}}{\min}$. Finally, use this dialysance to determine the pre‐filter RF rate: $\begin{array}{c} Q_{\mathrm{Pre}-\mathrm{RF}}=Q_{B}\cdot \frac{D_{\mathrm{Na}}-Q_{\mathrm{UF}}}{Q_{B}-D_{\mathrm{Na}}}=200\cdot \frac{17-0}{200-17}\approx \end{array}$ $\begin{array}{c} 18.6\;\frac{\text{ml}}{\min}=1.1\;\frac{L}{h} \end{array}$. Here, the UF rate is zero, so that calls for Equation (11) which is the limiting case if the TBW is stable. If we plug ~1.1 L/h for $Q_{\mathrm{Pre}-\mathrm{RF}}$ into a secant method on Equation (11), do we get ${\left[\mathrm{Na}+K\right]}_{\mathrm{Pre}-\mathrm{RF}}=136$ mEq/L? It is close: 136.2132533… Why is it not 136 exactly? Because we rounded the intermediate values in the osmotherapy algorithm. Without rounding, the $Q_{\mathrm{Pre}-\mathrm{RF}}$ is accurately 1.11672668147… L/h. Plugging this value in gets us the 136 mEq/L *exactly* for ${\left[\mathrm{Na}+K\right]}_{\mathrm{Pre}-\mathrm{RF}}$. This exercise was not an accident. We could fabricate other numbers to arrive at NaAR, $D_{\mathrm{Na}}$, and $Q_{\mathrm{Pre}-\mathrm{RF}}$, and Equation (11) would always yield the identical value for ${\left[\mathrm{Na}+K\right]}_{\mathrm{Pre}-\mathrm{RF}}$, hinting at an essential equivalence between osmotherapy and the mixing paradigm.

### 3.3 Proof of equivalence

To perform the osmotherapy calculations without rounding, simply carry over the variables, which preserve exact values, to each subsequent step in the algorithm. The NaAR is taken to be equal to the URR. From urea kinetic modeling (Daugirdas, 2015; Gotch & Sargent, 1985), $\mathrm{URR}=1-e^{-k\cdot \frac{t}{{\mathrm{TBW}}_{0}}}$, where k is the “klearance” rate of urea in pre‐filter CVVH if the TBW is stable (zero UF). Plug this into $\begin{array}{c} {\left[\mathrm{Na}+K\right]}_{\mathrm{Pre}-\mathrm{RF}}={\left[\mathrm{Na}\right]}_{0}+\frac{{\left[\mathrm{Na}\right]}_{t}-{\left[\mathrm{Na}\right]}_{0}}{\text{NaAR}} \end{array}$ to get $\begin{array}{c} {\left[\mathrm{Na}+K\right]}_{\mathrm{Pre}-\mathrm{RF}}={\left[\mathrm{Na}\right]}_{0}+\frac{{\left[\mathrm{Na}\right]}_{t}-{\left[\mathrm{Na}\right]}_{0}}{1-e^{-k\cdot \frac{t}{{\mathrm{TBW}}_{0}}}} \end{array}$. Add the two terms over a common denominator: $\begin{array}{c} {\left[\mathrm{Na}+K\right]}_{\mathrm{Pre}-\mathrm{RF}}= \end{array}$ $\begin{array}{c} \frac{{\left[\mathrm{Na}\right]}_{0}\cdot \left(1-e^{-k\cdot \frac{t}{{\mathrm{TBW}}_{0}}}\right)}{1-e^{-k\cdot \frac{t}{{\mathrm{TBW}}_{0}}}}+\frac{{\left[\mathrm{Na}\right]}_{t}-{\left[\mathrm{Na}\right]}_{0}}{1-e^{-k\cdot \frac{t}{{\mathrm{TBW}}_{0}}}}=\frac{{\left[\mathrm{Na}\right]}_{0}\cdot \left(1-e^{-k\cdot \frac{t}{{\mathrm{TBW}}_{0}}}\right)+{\left[\mathrm{Na}\right]}_{t}-{\left[\mathrm{Na}\right]}_{0}}{1-e^{-k\cdot \frac{t}{{\mathrm{TBW}}_{0}}}}= \end{array}$ $\begin{array}{c} \frac{{\left[\mathrm{Na}\right]}_{0}-{\left[\mathrm{Na}\right]}_{0}\cdot e^{-k\cdot \frac{t}{{\mathrm{TBW}}_{0}}}+{\left[\mathrm{Na}\right]}_{t}-{\left[\mathrm{Na}\right]}_{0}}{1-e^{-k\cdot \frac{t}{{\mathrm{TBW}}_{0}}}}=\frac{{\left[\mathrm{Na}\right]}_{t}-{\left[\mathrm{Na}\right]}_{0}\cdot e^{-k\cdot \frac{t}{{\mathrm{TBW}}_{0}}}}{1-e^{-k\cdot \frac{t}{{\mathrm{TBW}}_{0}}}} \end{array}$. What is k? For pre‐filter CVVH, k can be derived to be $\frac{Q_{B}\cdot Q_{\mathrm{Pre}-\mathrm{RF}}}{Q_{B}+Q_{\mathrm{Pre}-\mathrm{RF}}}$ (see Appendix A). Substituting this in for k,

(12) $${\left[\mathrm{Na}+K\right]}_{\mathrm{Pre}-\mathrm{RF}}=\frac{{\left[\mathrm{Na}\right]}_{t}-{\left[\mathrm{Na}\right]}_{0}\cdot e^{-\frac{Q_{B}\cdot Q_{\mathrm{Pre}-\mathrm{RF}}}{Q_{B}+Q_{\mathrm{Pre}-\mathrm{RF}}}\cdot \frac{t}{{\mathrm{TBW}}_{0}}}}{1-e^{-\frac{Q_{B}\cdot Q_{\mathrm{Pre}-\mathrm{RF}}}{Q_{B}+Q_{\mathrm{Pre}-\mathrm{RF}}}\cdot \frac{t}{{\mathrm{TBW}}_{0}}}}.$$

If the above equation can be recreated from Equation (11), then the equivalence is proved.

Equation (11) is first modified to fit the assumptions of zero UF and no post‐filter RF and no other inputs or outputs. Removing these modules and setting m=1, we can condense it to:

(13) $${\left[\mathrm{Na}\right]}_{t}={\left[\mathrm{Na}\right]}_{0}+\left\{1-e^{-\frac{Q_{B}\cdot Q_{\mathrm{Pre}-\mathrm{RF}}\cdot t}{\left(Q_{B}+Q_{\mathrm{Pre}-\mathrm{RF}}\right)\cdot {\mathrm{TBW}}_{0}}}\right\}\cdot \left\{{\left[\mathrm{Na}+K\right]}_{\mathrm{Pre}-\mathrm{RF}}\cdot \underset{=1}{\underbrace{Q_{\mathrm{Pre}-\mathrm{RF}}\cdot \left(1-\frac{Q_{\mathrm{Pre}-\mathrm{RF}}}{Q_{B}+Q_{\mathrm{Pre}-\mathrm{RF}}}\right)\cdot \frac{Q_{B}+Q_{\mathrm{Pre}-\mathrm{RF}}}{Q_{B}\cdot Q_{\mathrm{Pre}-\mathrm{RF}}}}}-{\left[\mathrm{Na}\right]}_{0}\right\}$$

Doing the algebra, we get a dramatic simplification in the second curly bracket:

(14) $${\left[\mathrm{Na}\right]}_{t}={\left[\mathrm{Na}\right]}_{0}+\left\{1-e^{-\frac{Q_{B}\cdot Q_{\mathrm{Pre}-\mathrm{RF}}}{Q_{B}+Q_{\mathrm{Pre}-\mathrm{RF}}}\cdot \frac{t}{{\mathrm{TBW}}_{0}}}\right\}\cdot \left\{{\left[\mathrm{Na}+K\right]}_{\mathrm{Pre}-\mathrm{RF}}-{\left[\mathrm{Na}\right]}_{0}\right\}.$$

Now, solve for ${\left[\mathrm{Na}+K\right]}_{\mathrm{Pre}-\mathrm{RF}}$:

(15) $${\left[\mathrm{Na}+K\right]}_{\mathrm{Pre}-\mathrm{RF}}=\frac{{\left[\mathrm{Na}\right]}_{t}-{\left[\mathrm{Na}\right]}_{0}\cdot e^{-\frac{Q_{B}\cdot Q_{\mathrm{Pre}-\mathrm{RF}}}{Q_{B}+Q_{\mathrm{Pre}-\mathrm{RF}}}\cdot \frac{t}{{\mathrm{TBW}}_{0}}}}{1-e^{-\frac{Q_{B}\cdot Q_{\mathrm{Pre}-\mathrm{RF}}}{Q_{B}+Q_{\mathrm{Pre}-\mathrm{RF}}}\cdot \frac{t}{{\mathrm{TBW}}_{0}}}}.$$

By inspection, Equation (15) is identical to Equation (12). QED.

### 3.4 Exploit general equation

Having validated our special case (steady TBW) against established osmotherapy (zero UF), we can use the generalized Equation (10) to explore even more complex scenarios. A hyponatremic patient needing CVVH is likely to be critically ill, on multiple drips and pressors, and is oliguric or anuric. These perturbations to the balance of Na/K and water can occur simultaneously, and it takes a differential equation to wrangle the many moving parts, even before introducing CVVH. In the hurry to initiate life‐saving care, there may not be time to dilute the RF in a sterile fashion, or mistakes can be made. If an RF has to be used as is, other CVVH parameters can compensate.

**FIGURE 2:**
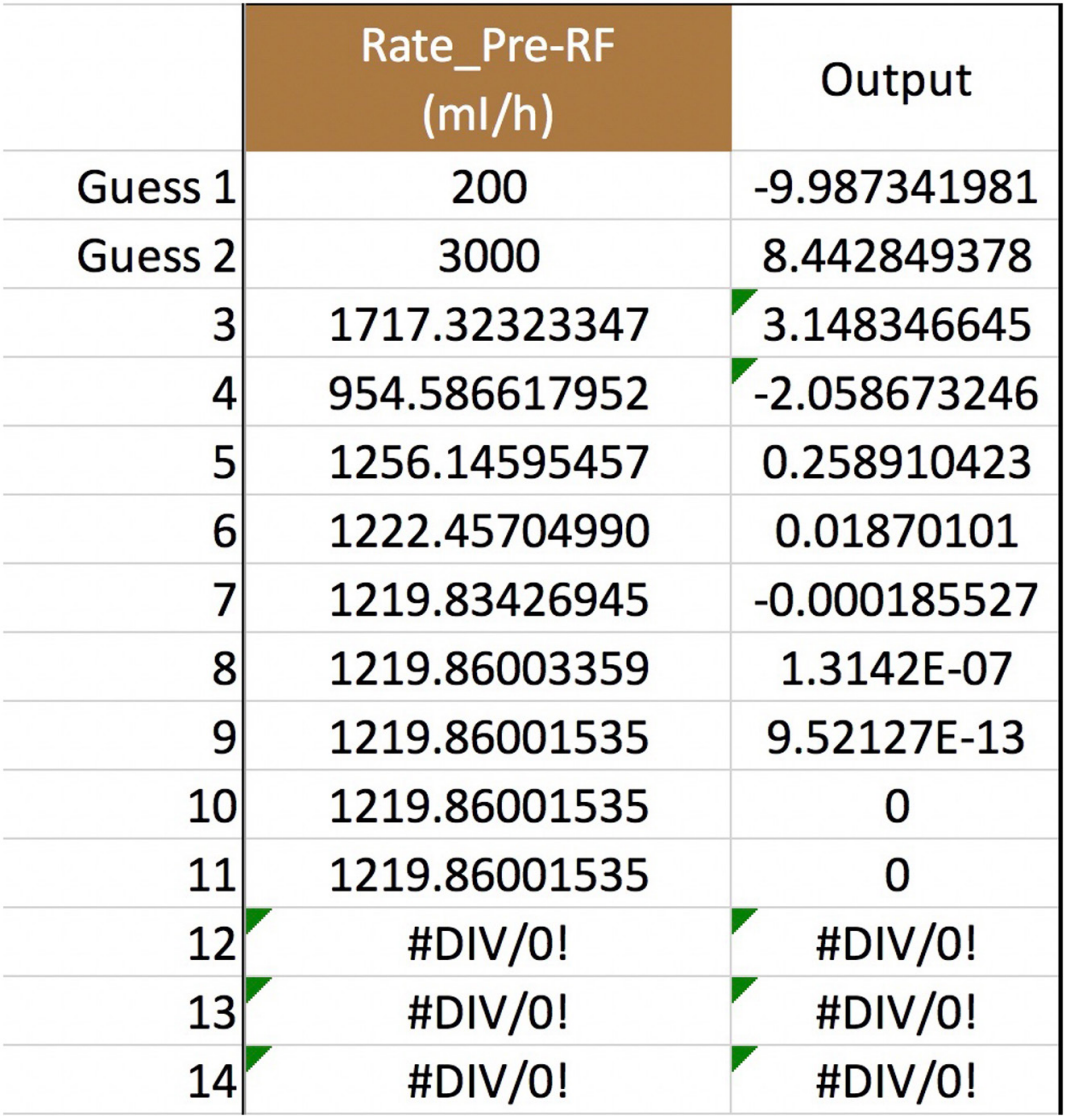
Secant method to calculate the pre‐filter RF rate. In this and the next few figures, ${\left[\mathrm{Na}\right]}_{0}=116$, ${\left[\mathrm{Na}\right]}_{t}=124$ mEq/L, t=24 h, ${\mathrm{TBW}}_{0}=48$ L, $Q_{B}=200$ ml/min, $Q_{\mathrm{UF}}=125$ ml/h, m=1, b=0, ${\left[\mathrm{Na}+K\right]}_{\text{In}}=0$, $Q_{\text{In}}=83.\bar{3}$ ml/h, and ${\left[\mathrm{Na}+K\right]}_{\mathrm{Pre}-\mathrm{RF}}=144$ mEq/L. There is no post‐filter RF or any other inputs or outputs. After the two initial guesses, the iterations converge on a value of ~1220 ml/h for the $Q_{\mathrm{Pre}-\mathrm{RF}}\text{.}$

To add more moving parts to our example patient (see p. 5), say that he has heart failure, is 3 kg above his dry weight, becomes hypotensive, and develops kidney failure with anuria. He gets pressors and other medicine drips in dextrose 5% water (D5W) that add up to 2 L/day. (Assume the medicines have no Na or K or tonicity.) The team wants CVVH to remove 3 L/day to put him into negative fluid balance, for the sake of the heart and lungs. Can the serum sodium still be raised from 116 to 124 mEq/L in 24 h? Well, the additional variables are ${\left[\mathrm{Na}+K\right]}_{\text{In}}=0\;\frac{\mathrm{mEq}}{L}$, $Q_{\text{In}}=\frac{2,000\;\text{ml}}{24\;h}$, and $Q_{\mathrm{UF}}=\frac{3,000\;\text{ml}}{24\;h}$. The RF is not altered, so ${\left[\mathrm{Na}+K\right]}_{\mathrm{Pre}-\mathrm{RF}}=144\;\frac{\mathrm{mEq}}{L}$. Using Equation (10) and a spreadsheet to do the secant method calculations, we find that dialing up the pre‐filter RF rate, $Q_{\mathrm{Pre}-\mathrm{RF}}$, from 1200 to 1220 ml/h will work (Figure 2). To check the root‐finding calculation, plug the non‐rounded value of 1219.86001535… ml/h for $Q_{\mathrm{Pre}-\mathrm{RF}}$ into Equation (10) to see that ${\left[\mathrm{Na}\right]}_{24\;h}=124$ mEq/L.

### 3.5 Flexibility and impossibility

**FIGURE 3:**
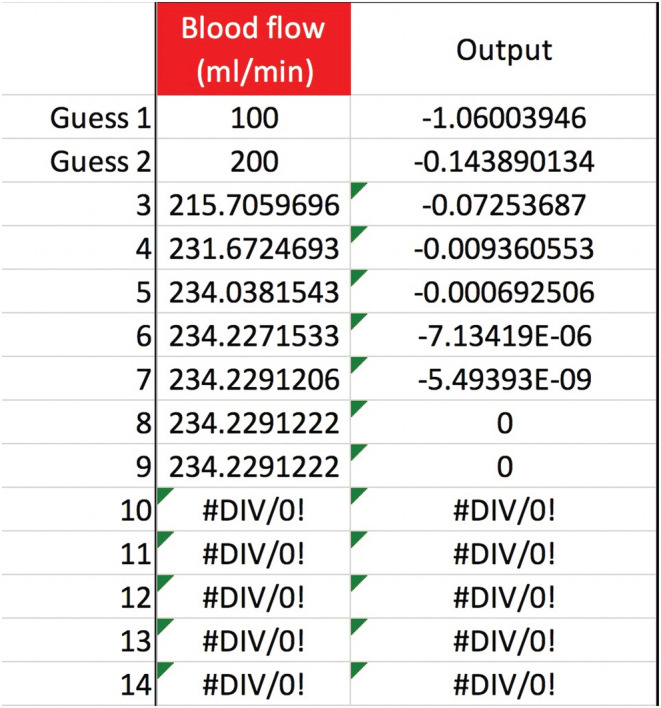
Secant method to calculate the blood flow rate. The variables have the same values as in Figure 2 (except $Q_{B}$, of course). Additionally, let $Q_{\mathrm{Pre}-\mathrm{RF}}=1,200$ ml/h. The secant method will then converge to $Q_{B}\approx 234$ ml/min, the CVVH setting needed to get the serum sodium up to 124 mEq/L at the 24‐h mark.

**FIGURE 4:**
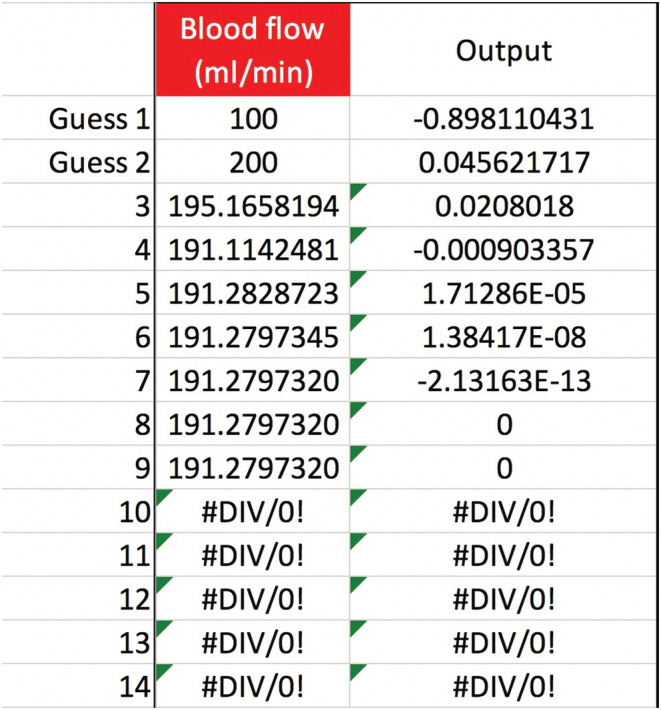
If the Edelman equation slope m were 1.05 instead of 1, then the secant method would calculate a $Q_{B}\approx 191$ ml/min instead. That makes sense. If the $\frac{\mathrm{Na}+K}{\mathrm{TBW}}$ changes the serum sodium by an extra 5%, then the required blood flow would be different.

**FIGURE 5:**
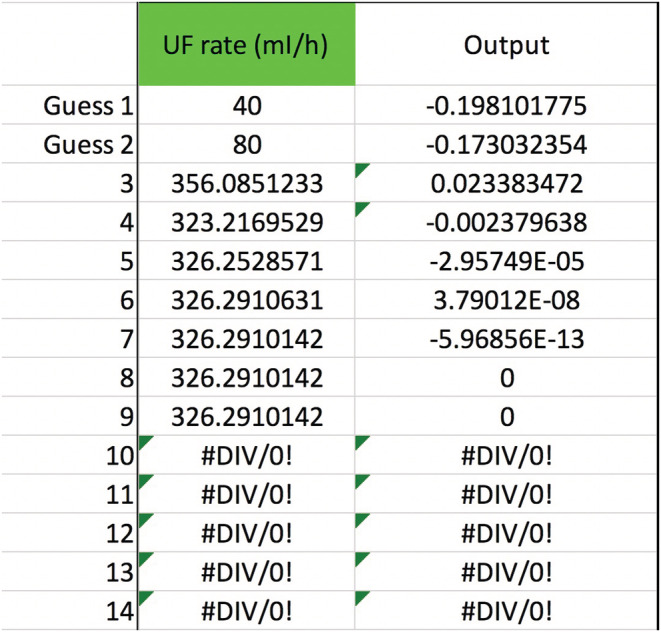
Secant method to calculate the net ultrafiltration rate. Revert back to m=1 and $Q_{B}=200$ ml/min and $Q_{\mathrm{Pre}-\mathrm{RF}}=1,200$ ml/h while all of the other variables are the same as in Figure 2 (except $Q_{\mathrm{UF}}$, of course). Now the secant method will produce a $Q_{\mathrm{UF}}\approx 326$ ml/h.

**FIGURE 6:**
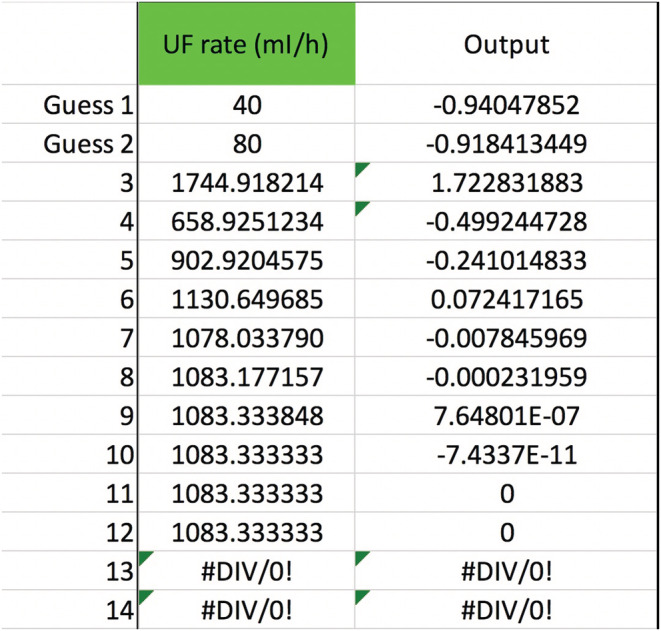
Secant method to calculate the net ultrafiltration rate if the $Q_{\mathrm{Pre}-\mathrm{RF}}$ was reduced to 1100 ml/h while the rest of the variables stayed the same as in Figure 2. To get to an ${\left[\mathrm{Na}\right]}_{24\;h}=124$ mEq/l, the new $Q_{\mathrm{UF}}$ would have to be $1,083.\bar{3}$ ml/h, which is clinically impossible to sustain.

**FIGURE 7:**
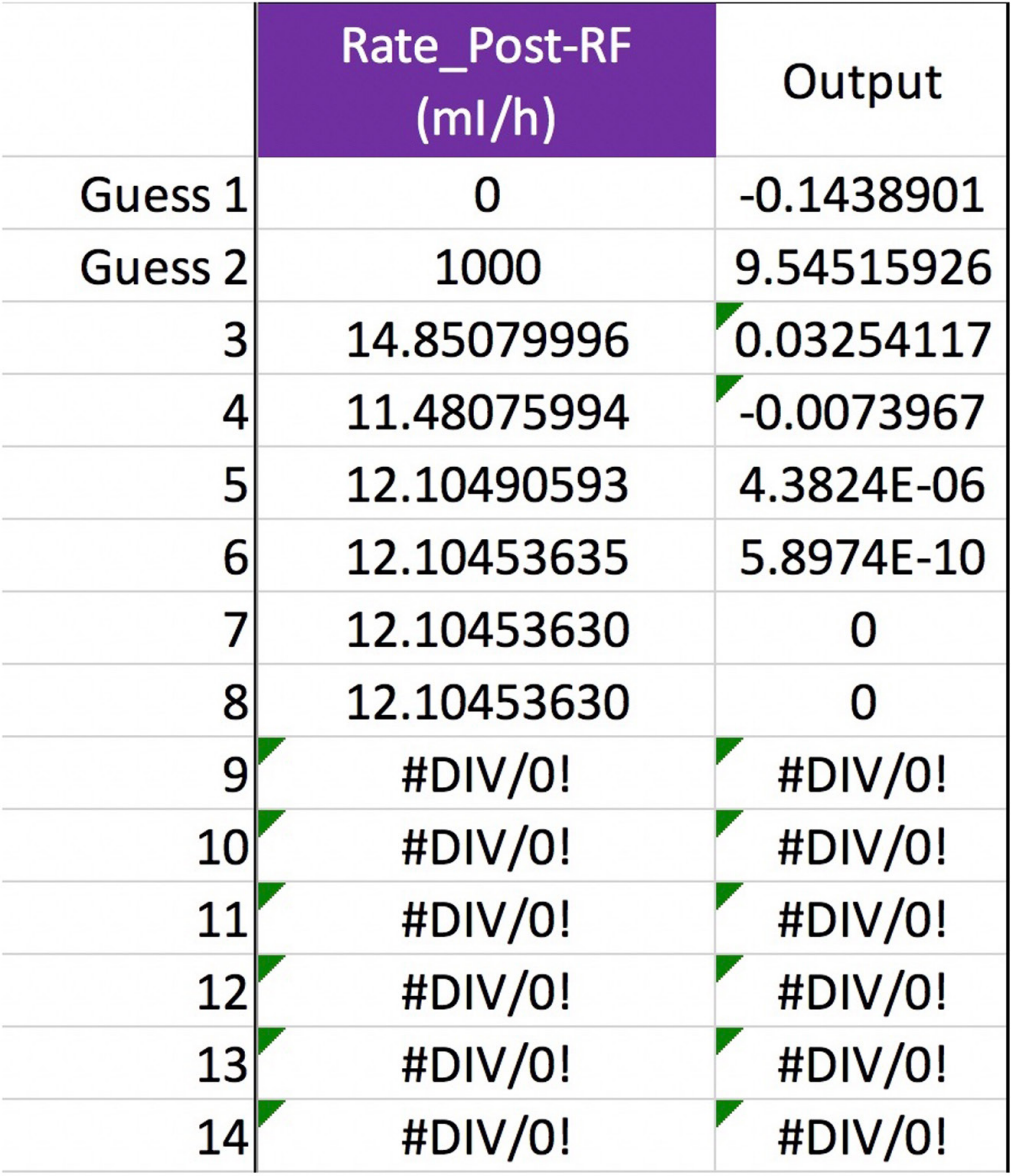
Secant method to calculate a post‐filter RF rate. Keep the variables as in Figure 2 plus $Q_{\mathrm{Pre}-\mathrm{RF}}=1,200$ ml/h. If a doctor wanted to add 0.9% saline as a post‐filter RF, then the secant method would say to infuse it at ~12 ml/h.

**FIGURE 8:**
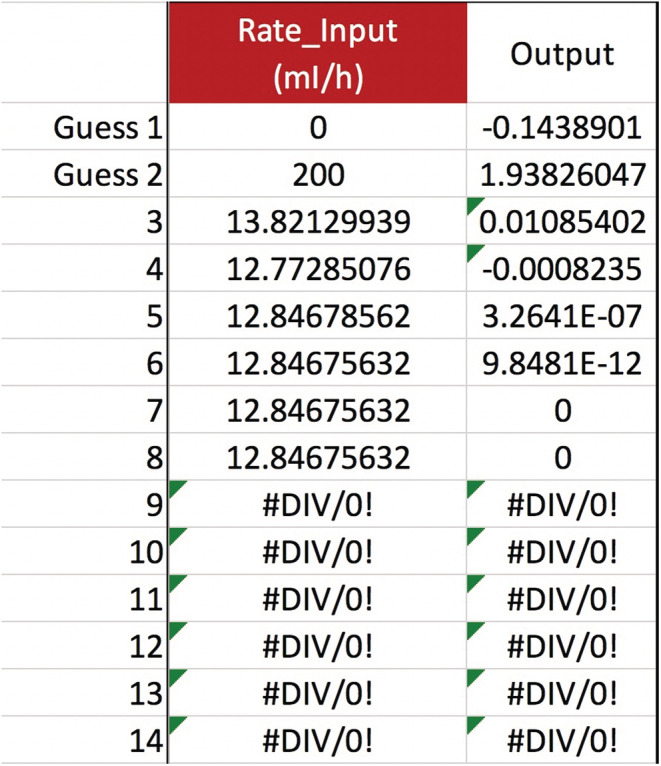
If the 0.9% saline were to be given in a peripheral intravenous line instead, while all else stayed the same as in Figure 7, then the secant method would say to infuse it at ~13 ml/h.

**FIGURE 9:**
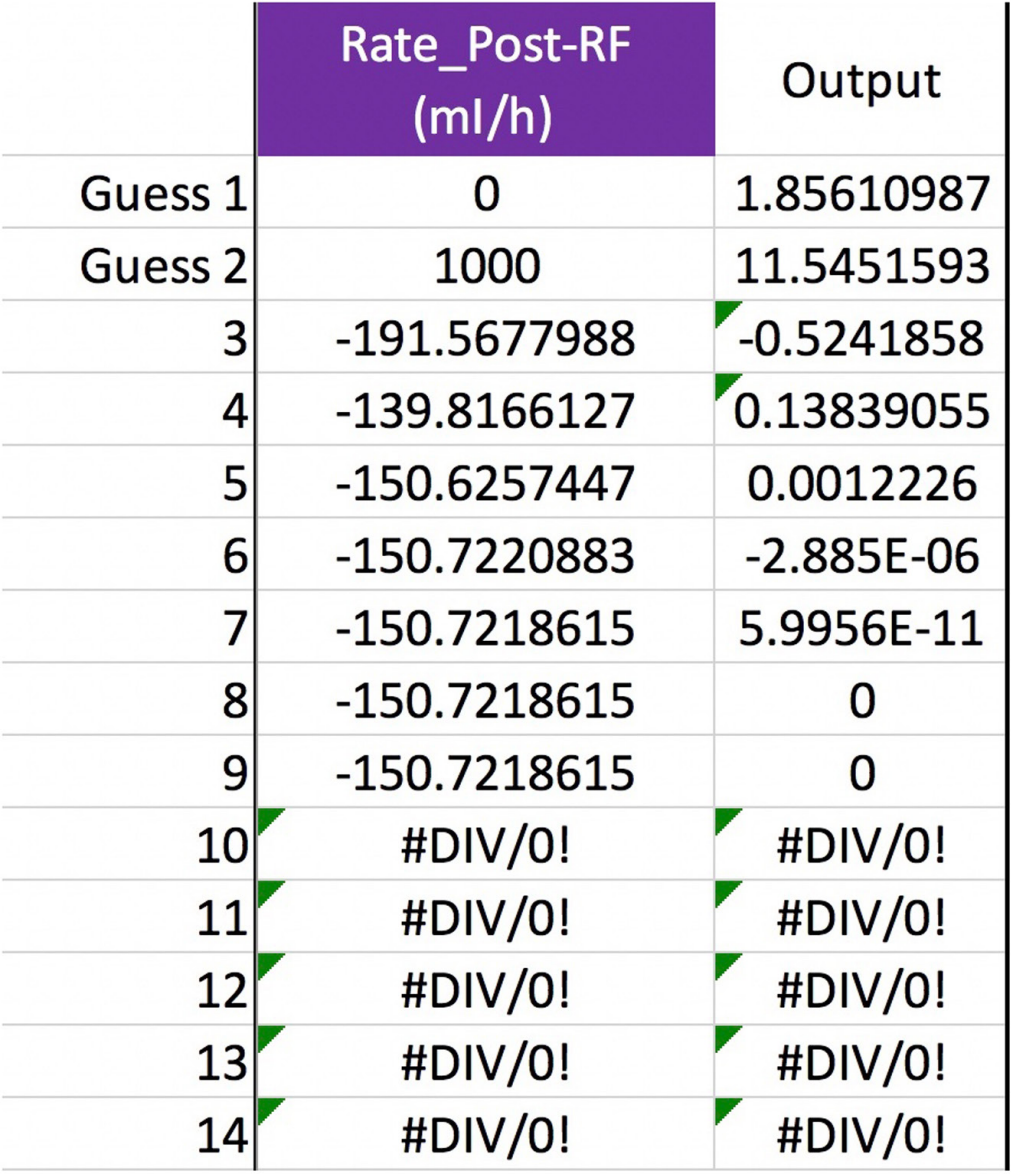
Aim for a more conservative $\Delta \left[\mathrm{Na}\right]$ of +6 instead of +8 mEq/L in 24 h. Keeping everything else the same as in Figure 7, the post‐filter 0.9% saline would be infused at about a *negative* 151 ml/h, according to the secant method. While impossible, the negative rate means that 0.9% saline should be extracted, the *opposite* of infused, from the CVVH circuit.

**FIGURE 10:**
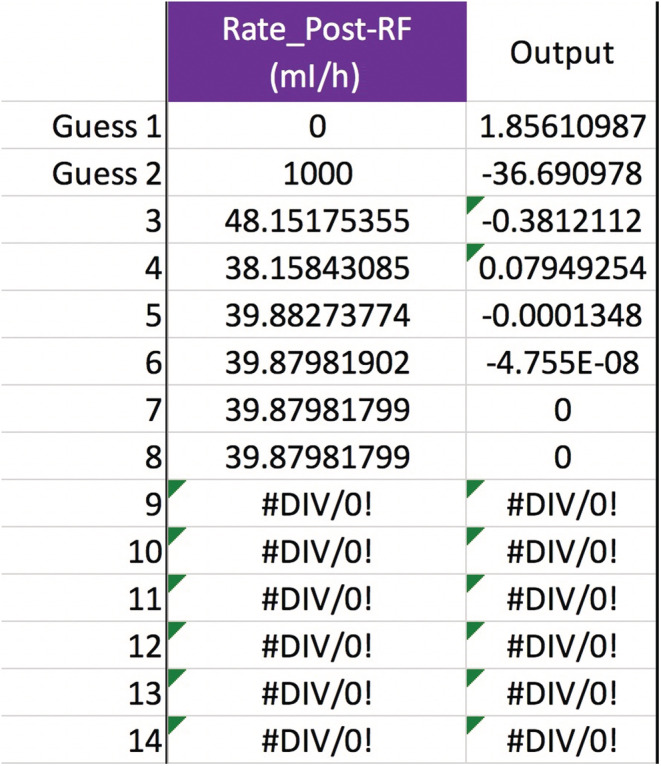
Going with a conservative sodium correction rate but keeping the other variables the same as in Figure 7 (except ${\left[\mathrm{Na}+K\right]}_{\text{Post}-\mathrm{RF}}$), we could infuse dextrose 5% water as a post‐filter RF instead of 0.9% saline. Now the secant method would yield a realistic rate of 40 ml/h.

Any of the variables in Equation (10), not just $Q_{\mathrm{Pre}-\mathrm{RF}}$, can be solved for to see how the [sodium] can be corrected safely. Obviously, not all variables are under a doctor's control, such as ${\left[\mathrm{Na}\right]}_{0}$, ${\mathrm{TBW}}_{0}$, or outputs like urine. But the variables that doctors do control afford a lot of flexibility in treatment. Adjusting the blood flow rate in CVVH is routine. The new calculated value is a $Q_{B}=234$, up from 200 ml/min but still quite manageable (Figure 3). If future studies reveal that the Edelman equation slope is really 1.05, then the $Q_{B}$ should be lowered to 191 ml/min (Figure 4). Back to m=1, what about the UF rate? That would have to increase to a $Q_{\mathrm{UF}}=326.3$ ml/h, or about 7.83 L/day which may or may not be doable (Figure 5). If the pre‐filter RF rate had been 1100 ml/h, close to what was calculated in **Gold standard** (see p. 5), then $Q_{\mathrm{UF}}$ would have to be 1083 ml/h, which is an impossible loss of 26 L/day (Figure 6). Back to $Q_{\mathrm{Pre}-\mathrm{RF}}=1,200$ ml/h, to assist in the correction of sodium, how about infusing 0.9% saline as a *post*‐filter RF? The $Q_{\text{Post}-\mathrm{RF}}$ would be a mere 12 ml/h (Figure 7). On the other hand, if 0.9% saline is infused in an IV line that is separate from CVVH, then the $Q_{\text{In}}$ would be closer to 13 ml/h (Figure 8). What if we were more cautious and aimed for a sodium increase of 6 mEq/L in 24 h? Setting ${\left[\mathrm{Na}\right]}_{t}=122$ mEq/L gives a $Q_{\text{Post}-\mathrm{RF}}$ of −151 ml/h (Figure 9). The negative sign is telling us to do the opposite of infuse, i.e., *remove* 0.9% saline from the CVVH line, which certainly is not feasible. But if the post‐filter RF were changed to D5W, then the $Q_{\text{Post}-\mathrm{RF}}$ would be a realistic 40 ml/h (Figure 10). These are some of the scenarios and parameter tweaks that can be explored.

## 4 DISCUSSION

Changes in the balance of sodium, potassium, and water follow physical rules that are described by mathematics. We should use this predictive tool to prescribe an osmotherapy that achieves the therapeutic goal while safeguarding the patient. Though our equations are fairly comprehensive, not every feature is called for all of the time. When the unused variables are zeroed out, the main equation reduces to a simpler form that replicates the previous formulas in the field of sodium kinetics. That is a necessary check on the validity of our work. Having passed the “special case” test, Equation (10) can be used to tackle the more complex cases that inevitably occur in real life.

### 4.1 Present

There is an opportunity to improve upon current practices. When faced with a low sodium that needs to be corrected slowly by CVVH, doctors often do a back‐of‐the‐envelope calculation. The teaching is that the serum sodium can be capped at the sodium concentration of the replacement fluid, assuring that the maximum rate of correction will not be exceeded. For example, a patient has an $\left[\mathrm{Na}\right]=112$ that should be raised to at most 118 mEq/L in the next 24 h while on CVVH. Blood flow is set at 200 ml/min, with no UF, and an RF will be given pre‐filter at 2000 ml/h to meet the recommended effluent flow rate of 20–25 ml/kg/h for an 80‐kg woman with a TBW of 44 L (Rosner & Connor Jr., 2018). If the RF's $\left[\mathrm{Na}+K\right]$ of 140 should be diluted to 118 mEq/L (cap) with a *post*‐filter D5W infusion, how fast should it be given? By trial and error, it was estimated that 400 ml/h is close enough, because the weighted average $\left[\mathrm{Na}+K\right]$ becomes $\frac{140\cdot 2,000+0\cdot 400}{2,000+400}=116.7$ mEq/L (Rosner & Connor Jr., 2018). Without trial and error, the direct way to calculate the D5W rate is to use algebra: $\frac{140\cdot 2,000+0\cdot x}{2,000+x}=118\to 140\cdot 2,000=118\cdot \left(2,000+x\right)\to x\approx 373$ ml/h. This is the same technique as a formula that is widely used to dilute the pre‐filter RF to a capping [Na]: $Q_{D5W}=\left(\frac{{\left[\mathrm{Na}+K\right]}_{\mathrm{Pre}-\mathrm{RF}}}{{\left[\mathrm{Na}\right]}_{\mathrm{cap}}}-1\right)\cdot Q_{\mathrm{Pre}-\mathrm{RF}}\to Q_{D5W}=\left(\frac{140}{118}-1\right)\cdot 2,000\approx 373$ ml/h (Lippold & Patel, 2020; Tinawi & Bastani, 2021).

### 4.2 Future?

How do these basic calculations compare to our model? Giving D5W at 373 ml/h to dilute the pre‐filter RF down to the capping [Na] of 118 mEq/L would make Equation (11) say that the ${\left[\mathrm{Na}\right]}_{t}$ increases from 112 to 116 mEq/L in 24 h. To get the rest of the way to 118 mEq/L, it would take ~48 more hours of CVVH with the capping method. A steeper sodium gradient, then, is needed to get the patient's [Na] from 112 to 118 mEq/L in 24 h. According to our method, the pre‐filter RF should be diluted to 121.2 mEq/L using D5W at 310 ml/h $\left(\frac{140\cdot 2,000+0\cdot 310}{2,000+310}\approx 121.2\right)$. That way, RF plus D5W at 2310 ml/h makes Equation (11) say that ${\left[\mathrm{Na}\right]}_{24\;h}=118$ mEq/L. As for infusing D5W *post*‐filter at 400 ml/h, Equation (11) would say that the ${\left[\mathrm{Na}\right]}_{24\;h}$ only gets up to 112.5 mEq/L. While certainly a safe [Na] correction rate, barely any progress has been made (+0.5 mEq/L). The minuscule increase could lead doctors to troubleshoot the CVVH machine, when the rudimentary calculation was to blame. The $Q_{\text{Post}-D5W}$ should be 260, not 400 ml/h. This rate dilutes less, providing the proper gradient between the RF and serum to bring the ${\left[\mathrm{Na}\right]}_{t}$ up to 118 mEq/L by the 24‐h mark (like an express train passing a station right at the scheduled time). Beyond 24 h, the ${\left[\mathrm{Na}\right]}_{t}$ would continue to rise, so the $Q_{\text{Post}-D5W}$ should be calculated anew, for example, to go from 118 to 124 mEq/L in the next 24 h. (Answer: lower $Q_{\text{Post}-D5W}$ to 167 ml/h.) Though it seems logical to create a slightly steeper gradient between the patient and the RF, our proposed practice is not yet backed by clinical testing, and it may not be better than the capping method. Until empirical data support our approach, it is theoretical.

### 4.3 Flow rate: Blood or plasma?

A difference between our mixing model and classic osmotherapy is the choice of blood flow rate vs. plasma flow rate. The latter $\left(Q_{P}\right)$ is used in osmotherapy, whereas the former $\left(Q_{B}\right)$ is used in Equation (10). To prove equivalence between the two (p. 6), we substituted in $Q_{B}$ wherever the osmotherapy equations call for $Q_{P}$. We think that $Q_{B}$ is correct. $Q_{B}$ is needed to calculate a weighted average $\left[\mathrm{Na}+K\right]$ when pre‐filter RF mixes with blood [Equation (3)]; after all, it is blood that flows in the CRRT tubing, not just plasma. Then, diluted blood is pushed across the hemofilter to become an ultrafiltrate. This effluent is aqueous, so its $\left[\mathrm{Na}+K\right]$ is well estimated by the diluted ${\left[\mathrm{Na}\right]}_{t}$. To make *serum* ${\left[\mathrm{Na}\right]}_{t}$ even closer to an aqueous concentration, we can divide ${\left[\mathrm{Na}\right]}_{t}$ by 0.93, which is roughly the fraction of serum that is aqueous (the fraction would be smaller in hyperlipidemic or hyperproteinemic states). In our mixing equations, the aqueous fraction can be folded into the slope and y‐intercept (multiply m and b by 0.93; Nguyen & Kurtz, 2004b). Overall, the debate between $Q_{B}$ and $Q_{P}$ would probably need to be settled by clinical research into which rate makes our model fit actual data better.

### 4.4 Options and adjustments

Periods other than 24 hours can be entered into the mixing equation. If we wanted to plan ahead in the Discussion example, we could have entered ${\left[\mathrm{Na}\right]}_{0}=112$, ${\left[\mathrm{Na}\right]}_{t}=124$ mEq/L, and t=48 h which still conforms to the correction goal of +6 mEq/L per 24 h. With everything else the same 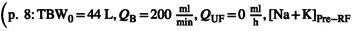 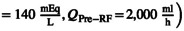, the post‐filter D5W should be infused at 191 ml/h. Fittingly, this rate falls between the two rates calculated earlier: (1) 260 ml/h for 112 to 118 mEq/L and (2) 167 ml/h for 118 to 124 mEq/L. Since the 191 ml/h value seems plausible, it can be tried, but we do not condone extending the time interval too much. Parameters can fluctuate, and the sodium trajectory does not go exactly as planned. The post‐filter D5W rate will have to be adjusted along the way, and the serum sodium needs to be frequently checked to monitor progress (Hanna et al., 2016; Sterns, 2016). If the sodium strays too far off the correction course, one option is to recalibrate the patient data. Maybe the ${\mathrm{TBW}}_{0}$ was significantly off, despite the best anthropometric efforts (Hume & Weyers, 1971; Mellits & Cheek, 1970; Watson et al., 1980). In that case, input the achieved $\frac{\Delta \left[\mathrm{Na}\right]}{\Delta t}$ and let the secant method deduce what the ${\mathrm{TBW}}_{0}$ should have been for the mixing model to fit reality. Then, use the revised ${\mathrm{TBW}}_{0}$ to calculate $Q_{\text{Post}-D5W}$ going forward.

The ability to solve for any of the variables in sodium kinetics greatly expands our toolbox. The practice may be to dilute the RF, but that requires a compounding pharmacist. Also, it breaks the bag's sterility, can introduce infection, and opens up the potential for procedural error (Tandukar et al., 2020). Instead, we can leave the RF intact and tweak another CVVH setting like $Q_{B}$ or $Q_{\mathrm{UF}}$ or $Q_{\mathrm{RF}}$. The secant method yields numerical answers that tell us what is possible and what is not (e.g., negative or excessive flow rate). Blood flow and RF rates may also be constrained by other criteria such as a minimum effluent rate or a maximum filtration fraction to avoid clotting the filter. Adding a peripheral IV drip is yet another option that Equation (10) can offer. In these ways, the mixing model gives us more prescriptive freedom than the osmotherapy algorithm.

### 4.5 Applicability

Though we have talked about chronic hyponatremia only, the sodium kinetic equations apply to the dysnatremias in general. If desired, one could calculate how to use CVVH to raise the sodium quickly in acute hyponatremia or to lower the sodium in hypernatremia, acute or chronic. These disorders' risks for osmotic demyelination or other neurological effect do not loom as large, if at all (Chauhan et al., 2019; Faber & Yee, 2020; George et al., 2018), so the mixing equation may not need to be consulted. One last issue to raise is the instantaneous mixing. This simplifying assumption does not simulate real life perfectly, but it makes the differential equation easier to solve. Still, it may not be too onerous to program in a mixing delay, mathematically.

### 4.6 Conclusions

It seems natural to model CVVH in the treatment of chronic hyponatremia as a mixing problem. By including so many sources of Na/K/H_2_O inputs and outputs into the differential equation, we hoped to make Equation (10) more powerful and versatile than the existing equations of sodium kinetics. At the same time, Equation (10) is just as precise as the old equations, because they are all based on the bedrock axioms of the field like the Edelman equation. Indeed, they were proved to be identical to each other in the special case when there are no IV fluids, urine output, or UF. The web of variables all working in concert can be intimidating, but the complexity is not unbearable, as the math is handled by software and clinical management is reduced to data entry. For motivated learners, the rationale of setting up a differential equation is the core concept to understand. The solving steps to yield Equation (10) are just rote procedures of math. With the equation plus a root‐finding technique, any of the variables can be accurately valued. That allows us to consider all of the different CVVH settings to get to a safe ${\left[\mathrm{Na}\right]}_{t}$. If a path is hazardous or not traversable, the math will warn us with an extreme value or a negative Q or $\left[\mathrm{Na}+K\right]$. For all its potential, our mixing theory should be clinically validated before it is widely used.

## AUTHOR CONTRIBUTIONS

Sheldon Chen: conception and design of study, mathematical derivations, interpretation of results, writing and revision of manuscript, and final approval of the manuscript. Jerry Yee: conceptualization of osmotherapy and the interpretation and checking of results. Robert Chiaramonte: confirmation of mathematical derivations, interpretation of results, and revision of manuscript.

## CONFLICT OF INTEREST

The authors have no conflicts of interest.

## ETHICAL STATEMENT

The patient cases are all hypothetical.

## APPENDIX A

**Introduction: Dye mixing problem** (from main text p. 2)

Define variables:

$C_{t}$: concentration of dye in the tank as a function of time.

$V_{t}$: volume of dye‐water mixture in the tank as a function of time.

Tank has 90 gallons of water to start with and increases by a net of 0.5 gallons per minute, so:

$$V_{t}=90+\frac{1}{2}t$$

Amount of dye at any given time is $C_{t}\cdot V_{t}$, so the instantaneous rate of change in the amount of dye can be expressed as $\frac{d}{dt}\left(C_{t}\cdot V_{t}\right)$. This must equal the rate of dye being pumped in minus the rate of dye being drained out. The rate of dye coming into the tank is:

$${\mathrm{Dye}}_{\text{In}}={\left[\mathrm{Dye}\right]}_{\text{In}}\cdot Q_{\text{In}}=\frac{100\%}{\text{gallon}}\cdot 1\;\frac{\text{gallon}}{\min}=1\;\mathrm{per}\;\min$$

The rate of dye being drained is the tank’s current dye concentration, $C_{t}$, times the drainage rate, 0.5 gallon/min.

$${\mathrm{Dye}}_{\text{Out}}={\left[\mathrm{Dye}\right]}_{\text{Out}}\cdot Q_{\text{Out}}=C_{t}\cdot \frac{1}{2}\;\frac{\text{gallon}}{\min}=\frac{1}{2}C_{t}\;\mathrm{per}\;\min$$

Therefore, write the differential equation as:

$$\frac{d}{dt}\left(C_{t}\cdot V_{t}\right)=1-\frac{1}{2}C_{t}$$

Differentiate the left side using the product rule:

$$\frac{dC_{t}}{dt}\cdot V_{t}+C_{t}\cdot \frac{dV_{t}}{dt}=1-\frac{1}{2}C_{t}$$

Substituting in $V_{t}=90+\frac{1}{2}t$ and differentiating w.r.t. time so that $\frac{dV_{t}}{dt}=\frac{1}{2}$, the above becomes:

$$\frac{dC_{t}}{dt}\cdot \left(90+\frac{1}{2}t\right)+\frac{1}{2}C_{t}=1-\frac{1}{2}C_{t}$$

Bring all of the $C_{t}$s to the left side of the equation:

$$\frac{dC_{t}}{dt}\cdot \left(90+\frac{1}{2}t\right)+C_{t}=1$$

Divide both sides by $\left(90+\frac{1}{2}t\right)$ to make the coefficient of $\frac{dC_{t}}{dt}$ into 1:

$$\frac{dC_{t}}{dt}+\frac{1}{90+\frac{1}{2}t}\cdot C_{t}=\frac{1}{90+\frac{1}{2}t}$$

The integrating factor (I.F.) is calculated as:

$$\exp\left(\int \frac{1}{90+\frac{1}{2}t}\cdot dt\right)=\exp\left(\ln\left(90+\frac{1}{2}t\right)\cdot 2\right)={\left(90+\frac{1}{2}t\right)}^{2}$$

Multiply both sides by the I.F.

$$\frac{dC_{t}}{dt}\cdot {\left(90+\frac{1}{2}t\right)}^{2}+\left(90+\frac{1}{2}t\right)\cdot C_{t}=90+\frac{1}{2}t$$

By design, the left side can now be condensed into the derivative of a product:

$$\frac{d}{dt}\left[C_{t}\cdot {\left(90+\frac{1}{2}t\right)}^{2}\right]=90+\frac{1}{2}t$$

Set up the integration:

$$\int d\left[C_{t}\cdot {\left(90+\frac{1}{2}t\right)}^{2}\right]=\int \left(90+\frac{1}{2}t\right)dt$$

Integrate both sides and add an arbitrary constant of integration K:

$$C_{t}\cdot {\left(90+\frac{1}{2}t\right)}^{2}=90t+\frac{1}{4}t^{2}+K$$

To solve for K, use initial conditions at t=0, when $C_{0}=0$ (dye concentration was zero because the tank had no dye to begin with):

$$0\cdot {\left(90+\frac{1}{2}\cdot 0\right)}^{2}=90\cdot 0+\frac{1}{4}\cdot 0^{2}+K\to K=0$$

The time at which the 100‐gallon tank is full can be algebraically found as:

$$V_{t}=90+\frac{1}{2}t\to 100=90+\frac{1}{2}t\to t=20\;\min$$

Or, intuitively, 10 more gallons are needed to fill the tank, and the tank is filling up at half a gallon/minute, so it would take 20 min.

Plug t=20min into the equation of [dye] as a function of time:

$$C_{20}\cdot {\left(90+\frac{1}{2}\cdot 20\right)}^{2}=90\cdot 20+\frac{1}{4}\cdot 20^{2}\to C_{20}=\frac{90\cdot 20+\frac{1}{4}\cdot 20^{2}}{{\left(90+\frac{1}{2}\cdot 20\right)}^{2}}$$

Compute $C_{20}$:

$$C_{20}=0.19\;\text{or}\;19\%$$

**General solution to the differential equation for CRRT** (from main text p. 2–4)

Differential Equation (9) (from main text p. 4):

On the left side, use the product rule to expand the differentiation:

Isolate all of the ${\left[\mathrm{Na}\right]}_{t}$s on the left side of the equation:

Turn the coefficient of $\frac{d{\left[\mathrm{Na}\right]}_{t}}{dt}$ into 1 by multiplying both sides by $\frac{m}{{\mathrm{TBW}}_{0}+\left(Q_{\text{In}}-Q_{\mathrm{UF}}-Q_{\text{Out}}\right)\cdot t}$:

The above is in the format of a first‐order, linear differential equation, so calculate the integrating factor (I.F.):

Multiply both sides by this I.F.:

The left side can now be condensed into the derivative of a product:

Integrate both sides:

Anti‐differentiate and add an arbitrary constant of integration C to the right side:

To find the value of C, use initial conditions. Plug in t=0 and then ${\left[\mathrm{Na}\right]}_{t}$ becomes ${\left[\mathrm{Na}\right]}_{0}$:

〈Na0−m⋅b⋅QIn−QUF−QOutm+Na+KPre‐RF⋅QPre‐RF+Na+KPost‐RF⋅QPost‐RF+Na+KIn⋅QIn−Na+KPre‐RF⋅QPre‐RFQB+QPre‐RF⋅QPre‐RF+QPost‐RF+QUF−Na+KOut⋅QOut⋅QB+QPre‐RFQB+QPre‐RF⋅QIn−QUF−QOut+QB⋅QPre‐RF+QPost‐RF+QUF⋅m〉⋅TBW01+QB⋅QPre‐RF+QPost‐RF+QUF⋅mQB+QPre‐RF⋅QIn−QUF−QOut=C.

Substitute the integration constant C back in:Divide both sides by ${\left[{\mathrm{TBW}}_{0}+\left(Q_{\text{In}}-Q_{\mathrm{UF}}-Q_{\text{Out}}\right)\cdot t\right]}^{\left\{1+\frac{Q_{B}\cdot \left(Q_{\mathrm{Pre}-\mathrm{RF}}+Q_{\text{Post}-\mathrm{RF}}+Q_{\mathrm{UF}}\right)\cdot m}{\left(Q_{B}+Q_{\mathrm{Pre}-\mathrm{RF}}\right)\cdot \left(Q_{\text{In}}-Q_{\mathrm{UF}}-Q_{\text{Out}}\right)}\right\}}$ to solve for  Conceptually, the serum sodium as a function of time should start from the initial [sodium], so:The introduction of ${\left[\mathrm{Na}\right]}_{0}$ allows for some grouping of common multiplier terms:

Group some more using an mx+b motif:

Nat=Na0+1−TBW0TBW0+QIn−QUF−QOut⋅t1+m⋅QB⋅QPre‐RF+QPost‐RF+QUFQB+QPre‐RF⋅QIn−QUF−QOut⋅m⋅Na+KPre‐RF⋅QPre‐RF⋅1−QPre‐RF+QPost‐RF+QUFQB+QPre‐RF+Na+KPost‐RF⋅QPost‐RF+Na+KIn⋅QIn−Na+KOut⋅QOut+b⋅QIn−QUF−QOut⋅QB+QPre‐RFQB+QPre‐RF⋅QIn−QUF−QOut+m⋅QB⋅QPre‐RF+QPost‐RF+QUF−Na0.

The above is more or less Equation (10) in the text (p. 4). Equation (10) has ellipses to show the modular nature of the Ins and Outs.

To get Equation (11) when the TBW is stable, the limit as $\left(Q_{\text{In}}-Q_{\mathrm{UF}}-Q_{\text{Out}}\right)\to 0$ can be found:

$$\mathrm{Let}\;y={\left[\frac{{\mathrm{TBW}}_{0}}{{\mathrm{TBW}}_{0}+\left(Q_{\text{In}}-Q_{\mathrm{UF}}-Q_{\text{Out}}\right)\cdot t}\right]}^{\left[1+m\cdot \frac{Q_{B}\cdot \left(Q_{\mathrm{Pre}-\mathrm{RF}}+Q_{\text{Post}-\mathrm{RF}}+Q_{\mathrm{UF}}\right)}{\left(Q_{B}+Q_{\mathrm{Pre}-\mathrm{RF}}\right)\cdot \left(Q_{\text{In}}-Q_{\mathrm{UF}}-Q_{\text{Out}}\right)}\right]}$$

$$\ln y=\left[1+m\cdot \frac{Q_{B}\cdot \left(Q_{\mathrm{Pre}-\mathrm{RF}}+Q_{\text{Post}-\mathrm{RF}}+Q_{\mathrm{UF}}\right)}{\left(Q_{B}+Q_{\mathrm{Pre}-\mathrm{RF}}\right)\cdot \left(Q_{\text{In}}-Q_{\mathrm{UF}}-Q_{\text{Out}}\right)}\right]\cdot \ln\left[\frac{{\mathrm{TBW}}_{0}}{{\mathrm{TBW}}_{0}+\left(Q_{\text{In}}-Q_{\mathrm{UF}}-Q_{\text{Out}}\right)\cdot t}\right]$$

$$\ln y=\left[1+m\cdot \frac{Q_{B}\cdot \left(Q_{\mathrm{Pre}-\mathrm{RF}}+Q_{\text{Post}-\mathrm{RF}}+Q_{\mathrm{UF}}\right)}{\left(Q_{B}+Q_{\mathrm{Pre}-\mathrm{RF}}\right)\cdot \left(Q_{\text{In}}-Q_{\mathrm{UF}}-Q_{\text{Out}}\right)}\right]\cdot \ln\left[1+\frac{-\left(Q_{\text{In}}-Q_{\mathrm{UF}}-Q_{\text{Out}}\right)\cdot t}{{\mathrm{TBW}}_{0}+\left(Q_{\text{In}}-Q_{\mathrm{UF}}-Q_{\text{Out}}\right)\cdot t}\right]$$

Use the Maclaurin series for $\ln\left(1+x\right)$ about x=0, since $\left(Q_{\text{In}}-Q_{\mathrm{UF}}-Q_{\text{Out}}\right)$ is going to zero:

$$y=\exp\left[-m\cdot \frac{Q_{B}\cdot \left(Q_{\mathrm{Pre}-\mathrm{RF}}+Q_{\text{Post}-\mathrm{RF}}+Q_{\mathrm{UF}}\right)\cdot t}{\left(Q_{B}+Q_{\mathrm{Pre}-\mathrm{RF}}\right)\cdot {\mathrm{TBW}}_{0}}\right]=e^{-m\cdot \frac{Q_{B}\cdot \left(Q_{\mathrm{Pre}-\mathrm{RF}}+Q_{\text{Post}-\mathrm{RF}}+Q_{\mathrm{UF}}\right)\cdot t}{\left(Q_{B}+Q_{\mathrm{Pre}-\mathrm{RF}}\right)\cdot {\mathrm{TBW}}_{0}}}$$

With the limit for the exponential solved, the rest of Equation (10) as $\left(Q_{\text{In}}-Q_{\mathrm{UF}}-Q_{\text{Out}}\right)\to 0$ is straightforward:

The above is more or less Equation (11) in the text (p. 5). Again, Equation (11) has the ellipses.

**Urea “klearance” for pre‐filter CVVH** (from main text, *Proof of equivalence*, p. 6)

Differential equation setup:

In urea kinetic modeling, the urea clearance rate is traditionally denoted as k. As with sodium kinetics, the rate of change in the amount of urea is equal to the rates of urea coming in minus the rates of urea going out of its volume of distribution, which is taken to be total body water. Let us consider the simple case of TBW being stable. If the only input is the pre‐filter RF and the only output is the CVVH effluent and both of their rates are matched, then the UF rate is zero:

$$Q_{\mathrm{Pre}-\mathrm{RF}}=Q_{\mathrm{Eff}},\mathrm{so}\;Q_{\mathrm{UF}}=0$$

Using the mixing problem construct to guide the setup of the differential equation, we know that the amount of blood urea nitrogen is the ambient [BUN] as a function of time multiplied by the TBW, which is stable. Dividing this product by a time interval gives the rate of change in BUN:

$$\frac{d}{dt}\left({\left[\mathrm{BUN}\right]}_{t}\cdot {\mathrm{TBW}}_{0}\right)\;\text{or}\;\frac{d{\left[\mathrm{BUN}\right]}_{t}}{dt}\cdot {\mathrm{TBW}}_{0}$$

On the other side of the differential equation is the mass balance of the urea inputs and outputs. RF does not contain any urea, so the urea input rate is zero. The CVVH effluent comprises all of the urea output. After the pre‐filter RF mixes with blood in the tubing, the effluent’s weighted average [BUN] is:

$${\left[\mathrm{BUN}\right]}_{\mathrm{Eff}}=\frac{{\left[\mathrm{BUN}\right]}_{t}\cdot Q_{B}+0\cdot Q_{\mathrm{Pre}-\mathrm{RF}}}{Q_{B}+Q_{\mathrm{Pre}-\mathrm{RF}}}$$

Keeping in mind that $Q_{\mathrm{Eff}}=Q_{\mathrm{Pre}-\mathrm{RF}}$, the rate of urea output is:

$${\left[\mathrm{BUN}\right]}_{\mathrm{Eff}}\cdot Q_{\mathrm{Eff}}=\frac{{\left[\mathrm{BUN}\right]}_{t}\cdot Q_{B}}{Q_{B}+Q_{\mathrm{Pre}-\mathrm{RF}}}\cdot Q_{\mathrm{Pre}-\mathrm{RF}}$$

Putting it all together, the differential equation becomes:

$$\frac{d{\left[\mathrm{BUN}\right]}_{t}}{dt}\cdot {\mathrm{TBW}}_{0}=\underset{\text{In rate}}{\underbrace{0}}-\underset{\text{Out rate}}{\underbrace{\frac{{\left[\mathrm{BUN}\right]}_{t}\cdot Q_{B}}{Q_{B}+Q_{\mathrm{Pre}-\mathrm{RF}}}\cdot Q_{\mathrm{Pre}-\mathrm{RF}}}}$$

Solve this using separation of variables:

$$\frac{d{\left[\mathrm{BUN}\right]}_{t}}{{\left[\mathrm{BUN}\right]}_{t}}=-\frac{Q_{B}\cdot Q_{\mathrm{Pre}-\mathrm{RF}}}{\left(Q_{B}+Q_{\mathrm{Pre}-\mathrm{RF}}\right)\cdot {\mathrm{TBW}}_{0}}dt$$

Integrate both sides and add an arbitrary constant of integration C:

$$\int \frac{d{\left[\mathrm{BUN}\right]}_{t}}{{\left[\mathrm{BUN}\right]}_{t}}=-\frac{Q_{B}\cdot Q_{\mathrm{Pre}-\mathrm{RF}}}{\left(Q_{B}+Q_{\mathrm{Pre}-\mathrm{RF}}\right)\cdot {\mathrm{TBW}}_{0}}\cdot \int dt$$

$$\ln{\left[\mathrm{BUN}\right]}_{t}=-\frac{Q_{B}\cdot Q_{\mathrm{Pre}-\mathrm{RF}}\cdot t}{\left(Q_{B}+Q_{\mathrm{Pre}-\mathrm{RF}}\right)\cdot {\mathrm{TBW}}_{0}}+C$$

Solve for ${\left[\mathrm{BUN}\right]}_{t}$:

$${\left[\mathrm{BUN}\right]}_{t}=\exp\left[-\frac{Q_{B}\cdot Q_{\mathrm{Pre}-\mathrm{RF}}\cdot t}{\left(Q_{B}+Q_{\mathrm{Pre}-\mathrm{RF}}\right)\cdot {\mathrm{TBW}}_{0}}+C\right]\equiv C\cdot \exp\left[-\frac{Q_{B}\cdot Q_{\mathrm{Pre}-\mathrm{RF}}\cdot t}{\left(Q_{B}+Q_{\mathrm{Pre}-\mathrm{RF}}\right)\cdot {\mathrm{TBW}}_{0}}\right]$$

Solve for C using initial conditions at t=0 when ${\left[\mathrm{BUN}\right]}_{t}={\left[\mathrm{BUN}\right]}_{0}$:

$${\left[\mathrm{BUN}\right]}_{0}=C\cdot \exp\left[-\frac{Q_{B}\cdot Q_{\mathrm{Pre}-\mathrm{RF}}\cdot 0}{\left(Q_{B}+Q_{\mathrm{Pre}-\mathrm{RF}}\right)\cdot {\mathrm{TBW}}_{0}}\right]$$

$${\left[\mathrm{BUN}\right]}_{0}=C$$

Thus, the differential equation’s solution is:

$${\left[\mathrm{BUN}\right]}_{t}={\left[\mathrm{BUN}\right]}_{0}\cdot \exp\left[-\frac{Q_{B}\cdot Q_{\mathrm{Pre}-\mathrm{RF}}\cdot t}{\left(Q_{B}+Q_{\mathrm{Pre}-\mathrm{RF}}\right)\cdot {\mathrm{TBW}}_{0}}\right]$$

Everything in the exponent that is *not* $-\frac{t}{{\mathrm{TBW}}_{0}}$ must be the k:

$$k=\frac{Q_{B}\cdot Q_{\mathrm{Pre}-\mathrm{RF}}}{Q_{B}+Q_{\mathrm{Pre}-\mathrm{RF}}}$$

Appropriately for k as a clearance rate, its unit is that of a flow rate Q, i.e., a volume per time. This replicates the postulated expression for k in the main text (p. 6).
